# FTY720 inhibited proinflammatory cytokine release and osteoclastogenesis induced by *Aggregatibacter actinomycetemcomitans*

**DOI:** 10.1186/s12944-015-0057-7

**Published:** 2015-07-04

**Authors:** Hong Yu, Bethany A. Herbert, Michael Valerio, Leigh Yarborough, Li-Chien Hsu, Kelley M. Argraves

**Affiliations:** Department of Oral Health Sciences and the Center for Oral Health Research, Medical University of South Carolina, 173 Ashley Avenue, Charleston, SC 29425 USA; Clemson University, Clemson, SC 29634 USA; Department of Regenerative Medicine and Cell Biology, Medical University of South Carolina, 173 Ashley Avenue, Charleston, SC 29425 USA

**Keywords:** FTY720, Sphingosine-1-phosphate, Periodontitis, Cytokine, Osteoclastogenesis, *Aggregatibacter actinomycetemcomitans*

## Abstract

**Background:**

Periodontitis is a bacteria-driven inflammatory bone loss disease. Previous studies showed that the oral pathogen *Aggregatibacter actinomycetemcomitans* (*A. actinomycetemcomitans*) stimulated the generation of sphingosine 1 phosphate (S1P). In addition, S1P signaling regulated the migration of osteoclast precursors and affected osteoclastogenesis. Furthermore, treatment with FTY720 (also called fingolimod, a modulator of multiple S1P receptors) alleviated osteoporosis and suppressed arthritis in animals. This study determined the effect of FTY720 on proinflammatory cytokine production and osteoclastogenesis in murine bone marrow cells with or without *A. actinomycetemcomitans* stimulation.

**Methods:**

Murine bone marrow-derived monocytes and macrophages (BMMs) were treated with vehicle ethanol or FTY720, and were either unstimulated or stimulated for 0.5 to 6 h with *A. actinomycetemcomitans*. The protein levels of interleukin (IL)-1β, IL-6, and tumor necrosis factor (TNF)-α in the media of BMMs were quantified by enzyme-linked immunosorbent assay (ELISA). Protein expressions, including phosphorylated phosphoinositide 3-kinase (p-PI3K), p-Akt, p-extracellular signal-regulated kinase (p-ERK), PI3K, Akt, and ERK were evaluated by Western blot. In addition, murine bone marrow-derived pre-osteoclasts were treated with macrophage colony-stimulating factor (M-CSF) and receptor activator of nuclear factor kappa-B ligand (RANKL) for three days. Then the cells were treated with either vehicle or FTY720 and were either unstimulated or stimulated with *A. actinomycetemcomitans* for 4 to 24 h. Control cells were treated with M-CSF alone with or without bacterial stimulation. Osteoclasts were stained by tartrate-resistant acid phosphatase (TRAP) staining. The mRNA levels of osteoclastogenic factors, including nuclear factor of activated T-cells cytoplasmic calcineurin-dependent 1 (Nfatc1), cathepsin K (Ctsk), acid phosphatase 5 (Acp5), osteoclast-associated receptor (Oscar), and RANKL were quantified by quantitative real-time polymerase chain reaction (PCR).

**Results:**

FTY720 dose-dependently inhibited IL-1β, IL-6, and TNF-α protein levels induced by *A. actinomycetemcomitans* in BMMs compared with controls. Additionally, FTY720 attenuated p-PI3K, p-Akt, and p-ERK expressions induced by *A. actinomycetemcomitans*. Furthermore, FTY720 suppressed osteoclastogenesis in bone marrow-derived pre-osteoclasts with or without bacterial stimulation and reduced the mRNA levels of Nfatc1, Ctsk, Acp5, and Oscar, but not RANKL in bone marrow-derived pre-osteoclasts.

**Conclusion:**

FTY720 inhibited proinflammatory cytokine production and suppressed osteoclastogenesis, supporting FTY720 as a potential therapy for inflammatory bone loss diseases.

## Background

Periodontitis is a bacteria-driven inflammatory bone loss disease. *A. actinomycetemcomitans* is an oral pathogen associated with localized aggressive periodontitis. Oral bacterial pathogens initiate a host inflammatory response, leading to proinflammatory cytokine production, progressive alveolar bone loss, and subsequent tooth loss [1]. One of the hallmarks of periodontitis is inflammation-induced osteoclastogenesis. While observed, the mechanisms associated with the inflammatory bone loss response induced by oral pathogens have not been completely elucidated.

Previously, we showed that *A. actinomycetemcomitans* stimulated the generation of sphingosine-1-phosphate (S1P) in RAW 264.7 cells, a murine monocyte and macrophage cell line [2]. S1P is a bioactive sphingolipid, which can be generated in most mammalian cells by various stimuli [3]. Intracellular S1P can be exported to extracellular space by specific transporters. S1P binds to five G protein-coupled S1P receptors (S1PR1-5) on the plasma membrane initiating various cellular signaling pathways [4, 5]. S1P signaling plays an important role in regulating cell growth, proliferation, adhesion, chemotaxis, cytokine production, and bone homeostasis [4, 6–8].

S1P signaling affects the pathogenesis of many diseases, including inflammatory diseases, arthritis, and osteoporosis [9–11]. The synovial fluid of rheumatoid arthritis patients exhibits significantly higher levels of S1P than their non-inflammatory osteoarthritis counterparts [12]. Additionally, S1P signaling controls the migration of monocytes and macrophages (osteoclast precursors) from blood circulation to bone tissues [7, 8] and stimulates the generation of RANKL, an osteoclastogenic factor, which affects bone homeostasis [13]. Furthermore, high circulating S1P levels observed in postmenopausal women are positively correlated with their bone resorption markers [14]. However, the roles of S1P signaling in modulating proinflammatory cytokine release and osteoclastogenesis induced by oral pathogens has not been defined.

FTY720 {2-amino-2-[2-(4-octylphenyl) ethyl]-1,3-propanediol hydrochloride} was synthesized by structural modification of myriocin, a fungal metabolite from *Isaclaria sinclarii*, a traditional herb used in Eastern medicine [15]. FTY720 is phosphorylated to p-FTY720 by sphingosine kinase. P-FTY720 functions as a noncompetitive inhibitor of multiple S1PRs [16, 17]. P-FTY720 blocks S1P signaling by inducing the internalization and partial degradation of S1PRs [16]. FTY720 has been shown as a potent immune suppressant with low toxicity. It has been used in clinical trials to treat relapsing multiple sclerosis and prevent the rejection of renal transplant [18, 19]. Additionally, treatment with FTY720 alleviated ovariectomy-induced osteoporosis in mice [7] and attenuated arthritis in mice induced by arthrogenic anti-collagen II antibodies cocktail and lipopolysaccharide, as compared with control treatment [20].

Monocytes and macrophages are major sources of proinflammatory cytokines in chronic inflammatory diseases. During inflammatory pathogenesis, bacterial pathogens activate various cellular signaling cascades including phosphoinositide 3-kinase (PI3K)-Akt (also known as protein kinase B), mitogen-activated protein kinases (MAPKs), and nuclear factor kappa-B (NF-κB) pathways. The MAPKs include the extracellular signal-regulated kinase (ERK), c-Jun N-terminal kinase (JNK), and p38 MAPK. Activation of these signaling pathways results in proinflammatory cytokine release. Additionally, monocytes and macrophages are osteoclast precursors, which can fuse to form multinucleated mature osteoclasts leading to bone loss [21]. Osteoclastogenesis is regulated by cytokines, including M-CSF and RANKL, which are essential for osteoclastogenesis [22]. M-CSF is essential for the survival and proliferation of osteoclast progenitors and macrophages, while RANKL is important for the differentiation of osteoclasts [22]. Additionally, nuclear factor of activated T-cells cytoplasmic calcineurin-dependent 1 (Nfatc1) is considered the master transcription factor during osteoclast differentiation [23]. Nfatc1 regulates transcription of many osteoclastogenic genes, including cathepsin K (Ctsk), acid phosphatase 5 (Acp5), and osteoclast-associated receptor (Oscar) [24, 25]. Although previous studies demonstrated that FTY720 inhibited immune response and attenuated bone loss [7, 18–20], the mechanisms associated with the roles of FTY720 on modulating inflammatory diseases and bone loss diseases have not yet been completely clarified. In this study, we elucidated the mechanisms associated with the effects of FTY720 in proinflammatory cytokine production and osteoclastogenesis with or without *A. actinomycetemcomitans* stimulation.

## Results

### FTY720 dose-dependently inhibited IL-1β, IL-6, and TNF-α protein levels induced by *A. actinomycetemcomitans* in BMMs

**Fig. 1:**
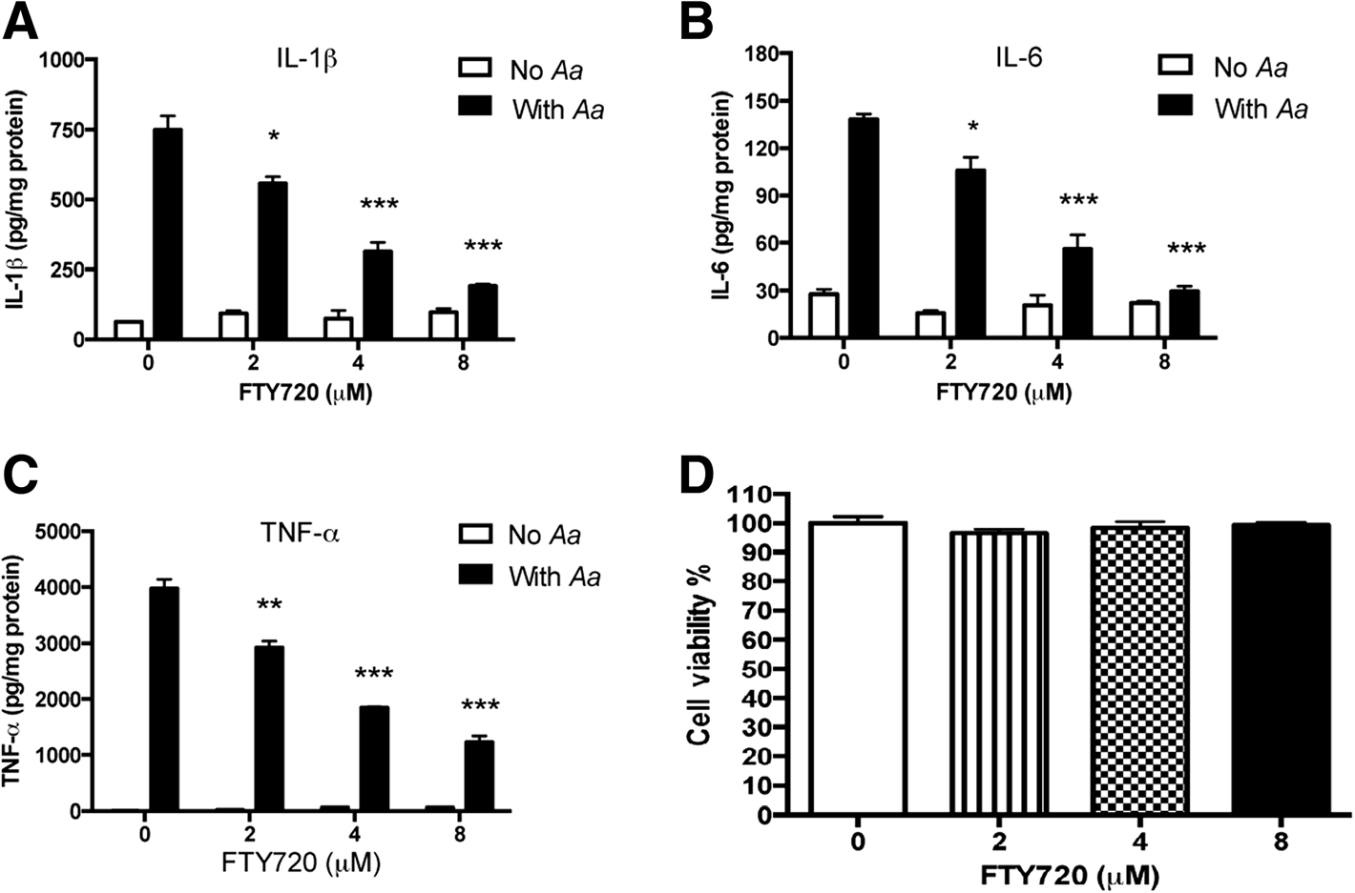
FTY720 dose-dependently inhibited IL-1β, IL-6, and TNF-α expressions induced by *A. actinomycetemcomitans* in BMMs. Murine BMMs were treated with vehicle (ethanol) or FTY720 (2 to 8 μM) for 30 min. Then the cells were either unstimulated or stimulated for 6 h with *A. actinomycetemcomitans* (*Aa*) (1.5 CFU/cell). **a** IL-1β, (**b**) IL-6, and (**c**) TNF-α protein levels in the cell culture media of BMMs were analyzed by ELISA. **d** Cell viability was tested in BMMs treated with vehicle or FTY720 (2 to 8 μM) for 8 h. Data are expressed as mean ± SEM (*n* = 3, *p < 0.05, **p < 0.01, ***p < 0.001)

Because FTY720 inhibited inflammatory response in previous *in vivo* studies [26], we hypothesized that FTY720 regulated the proinflammatory responses induced by *A. actinomycetemcomitans.* To test our hypothesis, BMMs derived from C57BL/6 mice were treated with either vehicle (ethanol) or FTY720 (2 to 8 μM) for 30 min. Then the cells were either unstimulated or stimulated with *A. actinomycetemcomitans* (1.5 CFU/cell) for 6 h. The protein levels of IL-1β, IL-6, and TNF-α in cell culture media were quantified. As shown in Fig. 1a-c, FTY720 significantly suppressed IL-1β, IL-6, and TNF-α expressions induced by *A. actinomycetemcomitans* in a dose-dependent manner compared with the control treatment*.* FTY720 (8 μM) decreased IL-1β by 74.5 %, IL-6 by 78.7 %, and TNF-α by 69.1 % induced by *A. actinomycetemcomitans* compared with the control treatment. FTY720 (4 μM) also reduced IL-1β by 58.3 %, IL-6 by 59.5 %, and TNF-α by 53.5 % induced by *A. actinomycetemcomitans* compared with the control treatment. FTY720 (2 to 8 μM) did not induce cell death in BMMs 8 h after treatment (Fig. 1d). These data supported that FTY720 suppressed the proinflammatory cytokine response induced by the oral pathogen *A. actinomycetemcomitans.*

### FTY720 attenuated p-PI3K, p-Akt, and p-ERK expressions induced by *A. actinomycetemcomitans* in BMMs

**Fig. 2:**
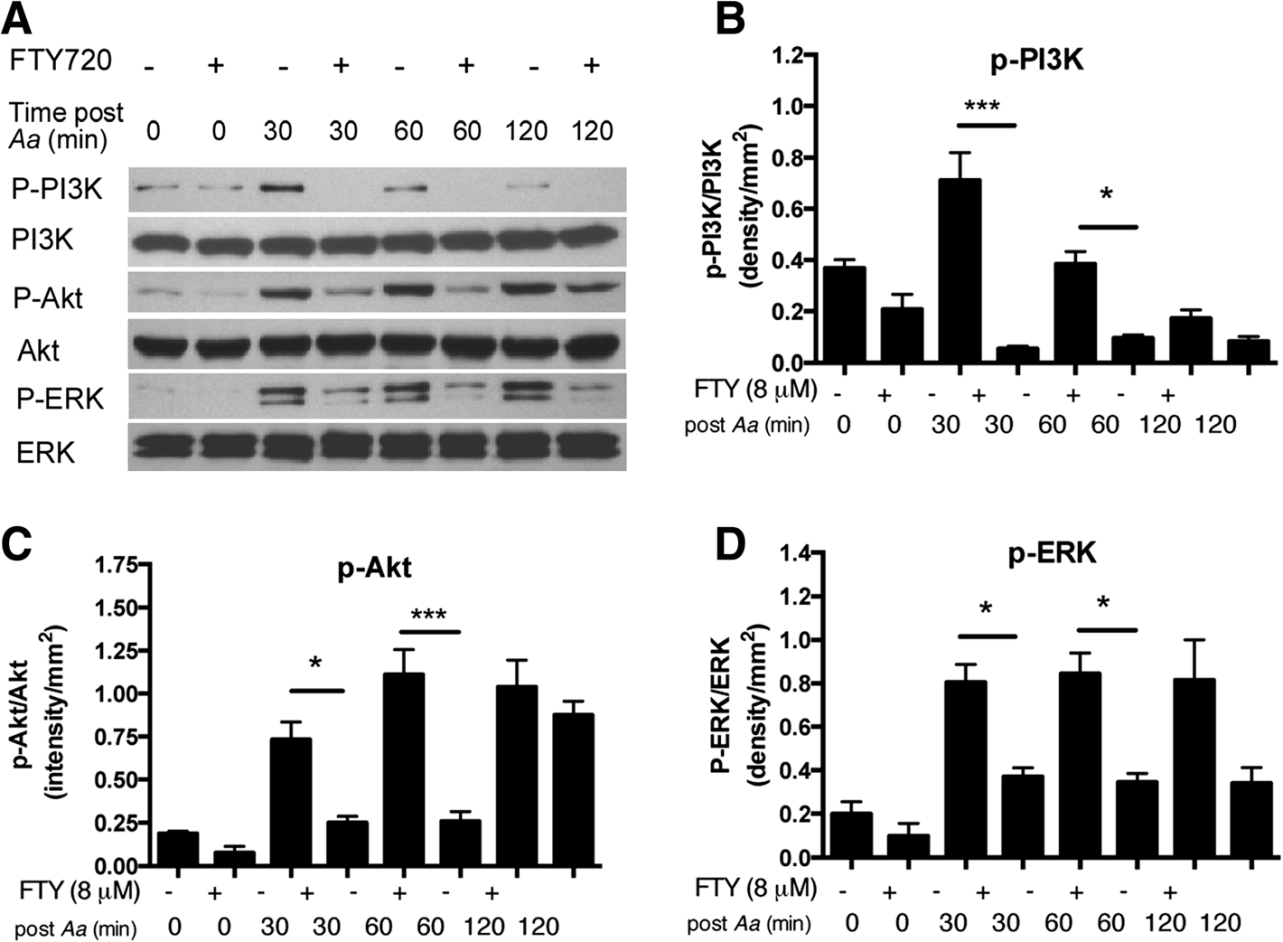
FTY720 attenuated p-PI3K, p-Akt, and p-ERK expressions induced by *A. actinomycetemcomitans* in BMMs. Murine BMMs were treated with vehicle (ethanol) or FTY720 (8 μM) for 30 min. Then the cells were either unstimulated or stimulated with *A. actinomycetemcomitans* (*Aa*) (1.5 CFU/cell) for 30 to 120 min. **a** P-PI3K, PI3K, p-Akt, Akt, p-ERK, and ERK expressions were evaluated by Western blot. **b** P-PI3K protein density, (**c**) P-Akt protein density, and (**d**) P-ERK protein density were analyzed by Quantity One Software and normalized by total protein expression, respectively. Data are expressed as mean ± SEM (*n* = 3, *p < 0.05, *** *p* < 0.001)

To further elucidate which signaling pathways were affected by FTY720 in regulating the immune response induced by *A. actinomycetemcomitans,* we performed Western blot assays in BMMs treated with vehicle (ethanol) or FTY720 (8 μM), with or without *A. actinomycetemcomitans* stimulation. As shown in Fig. 2a-d, FTY720 treatment decreased p-PI3K by 92.5 %, p-Akt by 65.9 %, and p-ERK by 54.0 % 30 min after bacterial stimulation compared with the control treatment. FTY720 reduced p-PI3K by 75.2 %, p-Akt by 76.9 %, and p-ERK by 59.1 % 60 min after bacterial stimulation compared with the control treatment. Additionally, FTY720 attenuated p-PI3K by 43.6 %, p-Akt by 59.2 %, and p-ERK by 50.0 % in cells without bacterial stimulation compared with the control treatment. The protein levels of p-NF-κB p65, p-JNK, p-p38 MAPK, and glyceraldehyde 3-phosphate dehydrogenase (GAPDH) were similar between FTY720-treated cells and vehicle-treated cells before or after bacterial stimulation (data not shown). These results supported that FTY720 specifically attenuated the PI3K, Akt, and ERK signaling pathways, which could contribute to the down-regulation of the proinflammatory cytokine response stimulated by *A. actinomycetemcomitans.*

### FTY720 suppressed osteoclastogenesis in bone marrow-derived pre-osteoclasts with or without bacterial stimulation

**Fig. 3:**
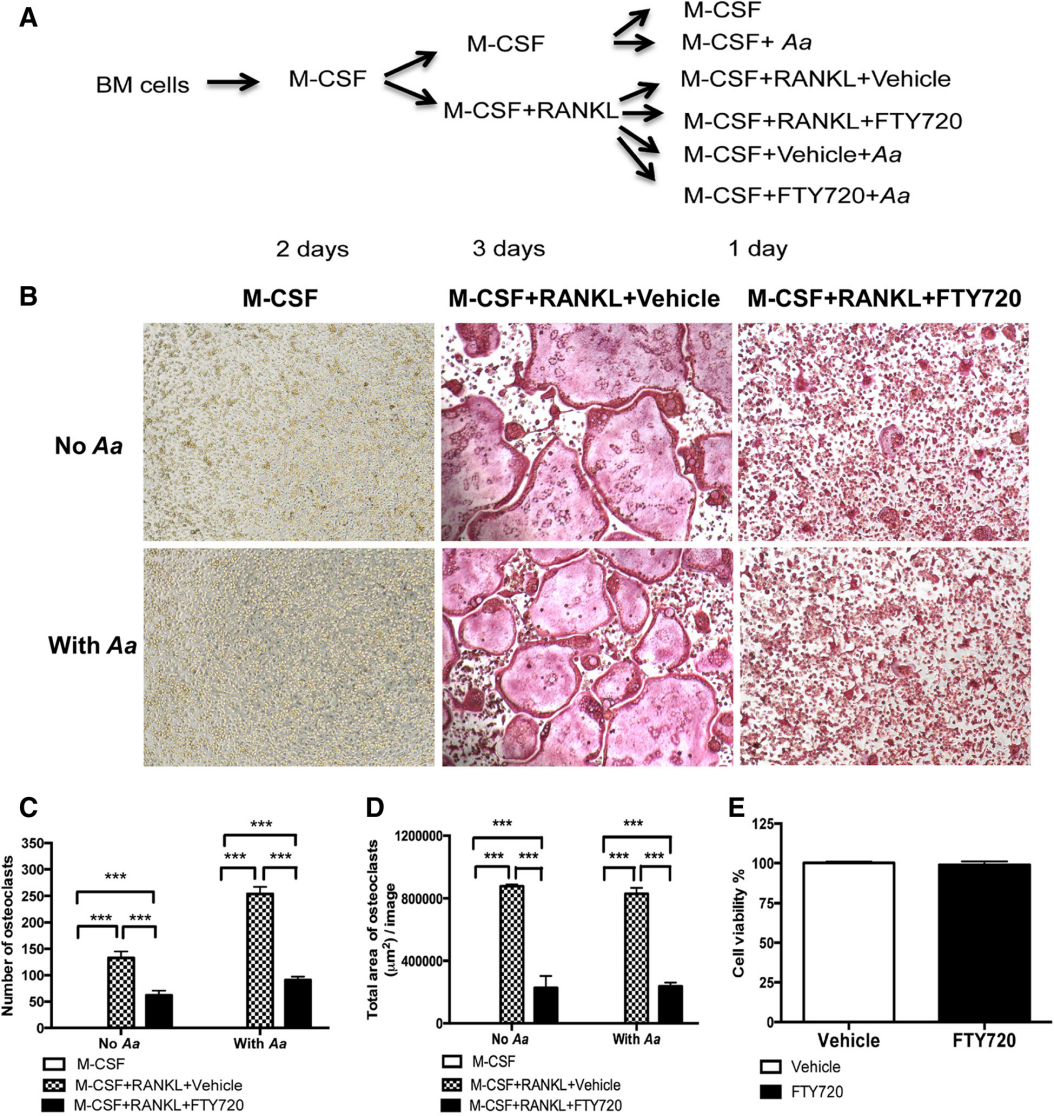
FTY720 suppressed osteoclastogenesis in bone marrow-derived pre-osteoclasts treated with or without *A. actinomycetemcomitans* (*Aa*) stimulation. **a** Bone marrow (BM) cells were treated as described in Methods. **b** Representative images show TRAP-stained cells with or without *A. actinomycetemcomitans* stimulation. Pictures were taken at 100× magnification. **c** Number of TRAP^+^ multinucleated (more than 3 nuclei) osteoclasts/well (96-well) were quantified. **d** Total areas for osteoclasts/image were quantified. **e** Cell viability was tested in bone marrow-derived pre-osteoclasts treated with vehicle or FTY720 (2 μM) for 24 h. Data are expressed as mean ± SEM (*n* = 4, ***p < 0.001)

Osteoclasts originate from the fusion of monocytes and macrophages [27]. It has been recognized that phosphoinositides signaling controls the activation of Nfatc1 and influences osteoclastogenesis [28]. Furthermore, proinflammatory cytokines promote the differentiation of osteoclasts [1]. Since FTY720 significantly inhibited PI3K signaling and attenuated proinflammatory cytokine expression induced by *A. actinomycetemcomitans*, we hypothesized that FTY720 could further inhibit osteoclastogenesis*.* To test our hypothesis, murine bone marrow cells were treated with M-CSF (50 μg/mL) for two days to allow bone marrow progenitor cells to differentiate into bone marrow-derived pre-osteoclasts. To determine if FTY720 could inhibit osteoclastogenesis induced by RANKL, bone marrow-derived pre-osteoclasts were treated with M-CSF (50 μg/mL) and RANKL (100 ng/mL) for three days; then the media were changed with fresh media containing M-CSF (50 μg/mL) and RANKL (100 ng/mL). Cells were treated with FTY720 (2 μM) or vehicle (ethanol) for 24 h (Fig. 3a). Additionally, to determine if FTY720 could attenuate osteoclastogenesis induced by *A. actinomycetemcomitans*, bone marrow-derived pre-osteoclasts were treated with M-CSF (50 μg/mL) and RANKL (100 ng/mL) for three days. To reduce the background of osteoclastogenesis induced by RANKL, the media were changed with media containing only M-CSF (50 μg/mL) without RANKL. The cells were treated for 30 min with vehicle or FTY720 (2 μM). Then the cells were either unstimulated or stimulated for 24 h with *A. actinomycetemcomitans* (0.5 CFU/cell), in the presence of FTY720 or vehicle (Fig. 3a). Control cells were treated with media containing only M-CSF (50 μg/mL) with or without bacterial stimulation. Osteoclasts were detected by tartrate-resistant acid phosphatase (TRAP) staining 24 h after FTY720 or vehicle treatment. As shown in Fig. 3b, there were no TRAP^+^ osteoclasts in cells treated with only M-CSF with or without bacterial stimulation. There were many TRAP^+^ multinucleated osteoclasts in cells treated with vehicle in the presence of both M-CSF and RANKL with or without bacterial stimulation. In contrast, FTY720 (2 μM) treatment decreased both size and number of TRAP^+^ multinucleated osteoclasts compared with vehicle groups. Quantification of the number of osteoclasts showed that there was a 1.9-fold increase of the number of osteoclasts in cells treated with M-CSF and RANKL stimulated with *A. actinomycetemcomitans* compared with cells treated with M-CSF and RANKL without bacterial stimulation (Fig. 3c). FTY720 treatment reduced the number of osteoclasts by 53.2 % in cells treated with M-CSF and RANKL without bacterial stimulation, and decreased the number of osteoclasts by 64.3 % in cells treated with M-CSF and RANKL with bacterial stimulation, as compared with vehicle controls, respectively (Fig. 3c). Quantification of total area of osteoclasts per image revealed that FTY720 reduced the area of osteoclasts by 74.2 % in cells treated with M-CSF and RANKL without bacterial stimulation, and FTY720 decreased the area of osteoclasts by 71.4 % in cells treated with M-CSF and RANKL with bacterial stimulation (Fig. 3d). FTY720 (2 μM) treatment for 24 h did not induce cell death in bone marrow-derived pre-osteoclast (Fig. 3e). These data demonstrated that FTY720 suppressed osteoclastogenesis in bone marrow-derived pre-osteoclasts treated with M-CSF and RANKL with or without bacterial stimulation.

### FTY720 significantly decreased the mRNA expressions of Nfatc1, Ctsk, Acp5, and Oscar in bone marrow-derived pre-osteoclasts with or without bacterial stimulation

**Fig. 4:**
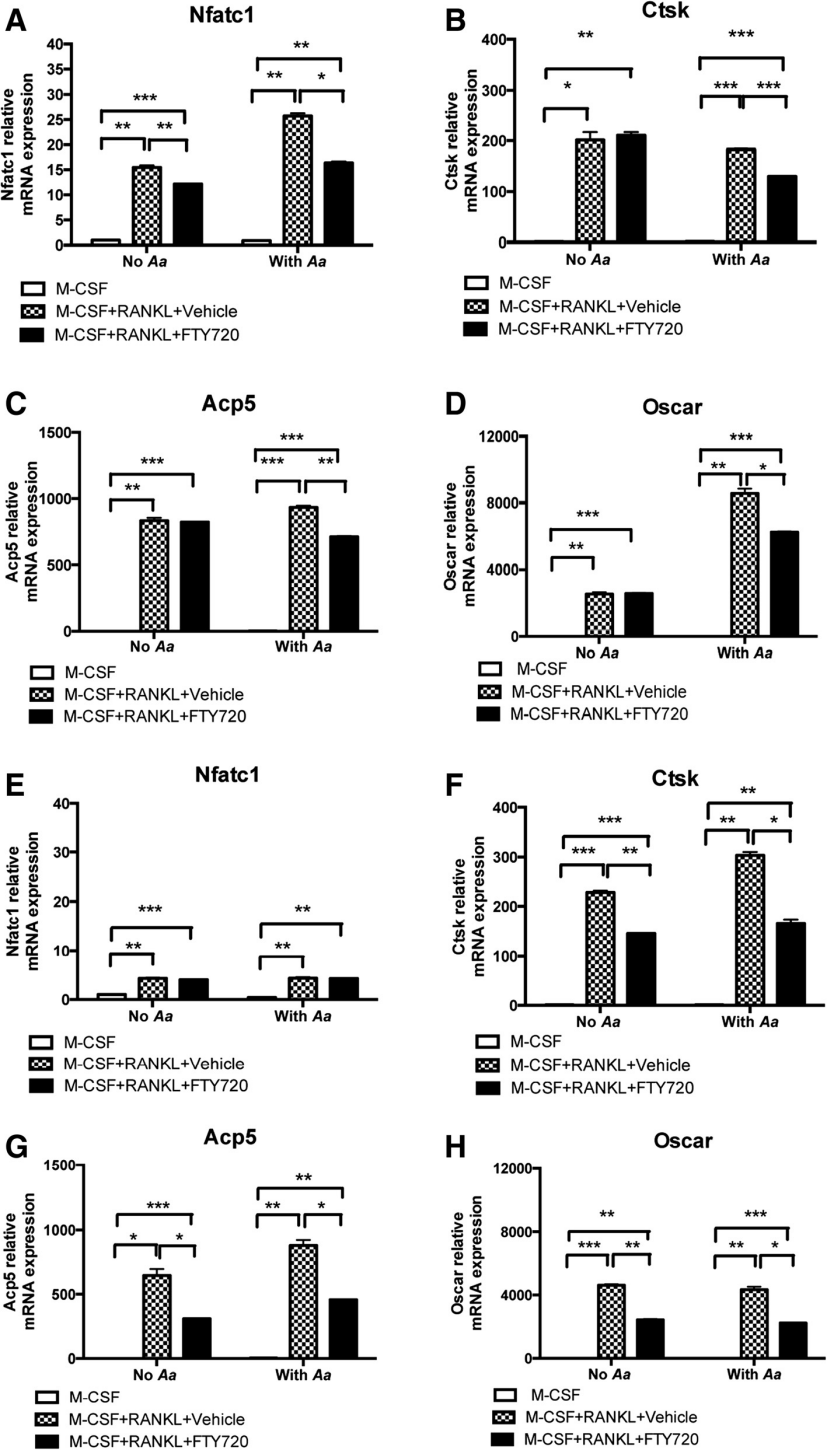
FTY720 significantly decreased Nfatc1, Ctsk, Acp5, and Oscar expressions in bone marrow-derived pre-osteoclasts with or without *A. actinomycetemcomitans* (*Aa*) stimulation. Bone marrow-derived pre-osteoclasts were treated as described in Methods. Cells were treated with vehicle (ethanol) or FTY720 (2 μM) for 30 min. Then the cells were either unstimulated or stimulated with *A. actinomycetemcomitans* (*Aa*) (0.5 CFU/cell) in the presence of vehicle or FTY720 for 4 h (**a**-**d**) or for 24 h (**e**-**h**). **a**, **e** Nfatc1 mRNA, (**b**, **f**) Ctsk mRNA, (**c**, **g**) Acp5 mRNA, and (**d**, **h**) Oscar mRNA levels were normalized by an endogenous control GAPDH expression and expressed as fold change compared with control group. Data are expressed as mean ± SEM (*n* = 3, *p < 0.05, **p < 0.01, ***p < 0.001)

To elucidate the mechanisms associated with the role of FTY720 in osteoclastogenesis, we analyzed the mRNA expressions of various kinds of osteoclastogenic factors, including Nfatc1, Ctsk, Acp5, Oscar, and RANKL in bone marrow-derived pre-osteoclasts treated for 4 or for 24 h with FTY720 (2 μM) or vehicle (ethanol), with or without *A. actinomycetemcomitans* stimulation. As shown in Fig. 4a-d, cells treated with both M-CSF and RANKL significantly increased the mRNA levels of Nfatc1, Ctsk, Acp5, and Oscar compared with those levels in cells treated with M-CSF alone. In cells treated with FTY720 for 4 h without bacterial stimulation (Fig. 4a), there was a 22.0 % significant reduction of Nfatc1 mRNA expression as compared with vehicle control. However, there were no significant differences of the mRNA levels of Ctsk, Acp5, and Oscar between FTY720-treated cells and vehicle-treated cells (Fig. 4b-d). In cells treated with FTY720 for 24 h without bacterial stimulation (Fig. 4. e-h), FTY720 significantly decreased the mRNA levels of Ctsk by 36.1 %, Acp5 by 51.5 %, and Oscar by 47.3 % compared with those levels in vehicle-treated cells. However, there was no significant difference in Nfatc1 mRNA expression between these two groups.

In cells treated with vehicle and stimulated with *A. actinomycetemcomitans* for 4 h (Fig. 4a-d), there was a 1.8-fold increase of Nfatc1 and a 3.6-fold increase of Oscar mRNA expression compared with those in cells without bacterial stimulation. FTY720 significantly reduced the mRNA levels of Nfatc1 by 36.5 %, Ctsk by 28.9 %, Acp5 by 23.6 %, and Oscar by 27.0 % compared with vehicle controls. In cells treated with vehicle stimulated with bacteria for 24 h (Fig. 4e-h), we observed a 1.3-fold increase of Ctsk and a 1.6-fold increase of Acp5 mRNA levels compared with those levels in cells treated with M-CSF and RANKL without bacterial stimulation. FTY720 significantly decreased the mRNA levels of Ctsk by 45.4 %, Acp5 by 47.7 %, and Oscar by 48.5 % compared with vehicle controls. However, there was no significant difference in Nfatc1 mRNA expression at 24 h between FTY720 treated-cells and vehicle-treated cells. These data supported that FTY720 decreased the mRNA expressions of osteoclastogenic factors, including Nfatc1, Ctsk, Acp5, and Oscar in bone marrow-derived pre-osteoclasts treated with M-CSF and RANKL with or without bacterial stimulation, which subsequently influenced the osteoclastogenesis.

## Discussion

Previous studies showed that FTY720, a modulator of multiple S1P receptors, exerted immunosuppressive activity and attenuated bone loss in mice [7, 18–20]. However, the mechanisms associated with the roles of FTY720 on modulating inflammatory diseases and bone loss diseases have not yet been completely elucidated. In this *in vitro* study, we demonstrated that FTY720 suppressed PI3K-Akt and ERK signaling pathways and attenuated IL-1β, IL-6, and TNF-α expressions induced by *A. actinomycetemcomitans.* Importantly, we first demonstrated that FTY720 suppressed osteoclastogenesis in bone marrow-derived pre-osteoclasts. Mechanistically, we demonstrated that FTY720 inhibited the expressions of osteoclastogenic factors, including Nfatc1, Ctsk, Acp5, and Oscar. Our study suggested that S1PR signaling pathways might be involved in the modulation of proinflammatory response induced by bacterial stimulation and may affect the osteoclastogenesis.

Previously, FTY720 was shown to exhibit an immunosuppressive effect *in vivo* [18, 19]. Early studies demonstrated that this effect was mainly caused by internalization and down-regulation of S1PR1 by FTY720 [26, 29], which resulted in the suppression of the egress of mature lymphocytes from the secondary lymphoid organs to peripheral blood and lymph [30]. In the current *in vitro* study, we demonstrated that FTY720 had a direct anti-inflammatory effect by suppressing IL-1β, IL-6 and TNF-α expression induced by the oral pathogen *A. actinomycetemcomitans*. This might be caused by the down-regulation of PI3K, Akt, and ERK signaling pathways by FTY720 in BMMs. Previous studies showed that human peripheral blood mononuclear cells (PBMCs) treated with a PI3K inhibitor, wortmannin, significantly inhibited LPS-induced chemokine C-X-C motif ligand 8 (CXCL8) and IL-6 release [31]. Additionally, PBMCs treated with pertussis toxin, a small G protein inhibitor, inhibited LPS-induced Akt phosphorylation and reduced the generation of CXCL8 and IL-6 [31]. Furthermore, murine macrophages treated with trametinib, a highly potent ERK inhibitor, significantly reduced LPS-induced TNF-α mRNA and protein secretion [32]. Since PI3K, Akt, and ERK signaling pathways were up-regulated by bacterial stimulation, and activation of these signaling pathways contributed to the production of proinflammatory cytokines, suppression of these signaling pathways by FTY720 might subsequently reduce IL-1β, IL-6 and TNF-α expressions induced by bacterial stimulation. Previously, Noda et al. [33] also demonstrated that FTY720 inhibited the production of LPS-induced proinflammatory cytokine IL-1β, IL-6, and TNF-α in microglia. In accordance with Noda et al., our study demonstrated that FTY720 suppressed IL-1β, IL-6 and TNF-α expressions induced by *A. actinomycetemcomitans.*

Although it is known that FTY720 is a modulator for multiple S1PRs, researchers continue to debate as to which S1PRs are regulated by p-FTY720. Early studies supported that p-FTY720 bound with higher affinity with S1PR1, 3, 4 and 5, and served as a S1P agonist [34, 35]. However, later studies demonstrated that p-FTY720 functioned as a noncompetitive inhibitor of multiple S1PRs [16, 17]. Graler et al. [16] demonstrated that FTY720 blocked S1P signaling through S1PR1, 2, and 5 by inducing their internalization and intracellular partial degradation without affecting S1PR3 or S1PR4. Another study showed that human monocyte-derived dendritic cells treated with both FTY720 and p-FTY720 resulted in decreased S1PR1 and S1PR4 levels [17]. Future studies need to determine which of the five S1PRs might play a role in regulating the inflammatory response stimulated by *A. actinomycetemcomitans.*

In addition to modulating inflammatory response, previous studies demonstrated that S1P signaling was critical in modulating bone homeostasis [7, 14]. Lee et al. [14] showed that S1P levels were significantly higher in postmenopausal women than those in the premenopausal women and men, and the higher S1P levels in postmenopausal women were positively correlated with their bone resorption marker. In contrast, blocking S1P signaling by FTY720 inhibited osteoporosis in mice with ovariectomy [7]. Ishii et al. [7] explained that the anti-osteoporotic role of FTY720 is mainly caused by the inhibition of the migration of osteoclast precursors from the circulation to bone tissues. In the current *in vitro* assay, we demonstrated that FTY720 suppressed the differentiation of osteoclasts and attenuated the expressions of osteoclastogenic factors, including Nfatc1, Ctsk, Acp5, and Oscar.

Osteoclastogenesis involves fusion of osteoclast precursors to form multinucleated mature osteoclasts. It has been recognized that membrane lipids, especially phosphoinositides, are key signaling molecules that regulate osteoclastogenesis [28]. Activation of PI3K triggers the Ca^2+^ release followed by activation of Nfatc1, a master transcription factor for osteoclastogenic gene regulation [23, 28]. In this study, we demonstrated that FTY720 attenuated p-PI3K levels in BMMs before or after bacterial stimulation compared with vehicle treatment. Previously, Graler et al. [16] showed that HTC_4_ cells (rat hepatoma cells) treated with FTY720 decreased calcium release. We observed significantly decreased mRNA levels of Nfatc1, Ctsk, Acp5, and Oscar in FTY720-treated cells with or without bacterial stimulation compared with those in the vehicle-treated cells. The down-regulation of these osteoclastogenic factors by FTY720 might be associated with the decreased p-PI3K and possibly reduced intracellular calcium levels in FTY720-treated cells before or after bacterial stimulation. Since RANKL induces osteoclastogenesis via activation of Nfatc1 [36], down-regulation of Nfatc1 by FTY720 could inhibit osteoclastogenesis induced by RANKL without bacterial stimulation. In addition, reducing IL-1β, IL-6 and TNF-α expressions induced by bacterial stimulation by FTY720 could further attenuate osteoclastogenesis. In this study, we observed significant reductions of Nfatc1 mRNA levels by FTY720 at 4 h after treatment compared with vehicle controls, while we did not observe this significant reduction of Nfatc1 mRNA levels at 24 h in FTY720-treated cells compared with vehicle controls with or without bacterial stimulation. This suggests that activation of Nfatc1 is an early event that might occur before the activation of Ctsk, Acp5 and Oscar.

A previous study showed that low doses of FTY720 (20 to 100 nM) did not inhibit osteoclastogenesis in BMMs treated with RANKL and M-CSF for 4 days [13]. In this study, FTY720 (2 μM) suppressed osteoclastogenesis in bone marrow-derived pre-osteoclasts treated with M-CSF and RANKL with or without bacterial stimulation. Our study suggested that it might require higher doses of FTY720 (≥2 μM) to suppress p-PI3K and Nfatc1 expressions. Ryu et al. [13] demonstrated that S1P enhanced the expression of RANKL in osteoblasts, and FTY720 (10 nM) was potent to suppress S1P-induced RANKL expression in osteoblasts. In addition, they showed that FTY720 (10 nM) inhibited osteoclastogenesis induced by S1P in a co-culture of BMMs and osteoblasts [13]. Because RANKL is mainly produced in osteoblasts and mesenchymal stem cells, we did not observe a significant difference in RANKL expression between FTY720-treated cells and vehicle-treated cells in this single culture of bone marrow-derived pre-osteoclasts. As FTY720 is a modulator of multiple S1PRs, future studies need to determine which of the five S1PRs play a major role in regulating PI3K pathway, calcium release, and the expressions of various osteoclastogenic factors, including RANKL, Nfatc1, Ctsk, Acp5, and Oscar.

## Conclusions

FTY720 inhibited proinflammatory cytokine production in BMMs and suppressed osteoclastogenesis in bone marrow-derived pre-osteoclasts with or without *A. actinomycetemcomitans* stimulation, supporting FTY720 as a potential therapy for inflammatory bone loss diseases.

## Methods

### Murine bone marrow-derived monocytes and macrophages (BMMs) and reagents

Six to eight-week-old male C57BL/6 mice were purchased from Jackson Laboratory (Bar Harbor, ME, USA). Bone marrow cells were harvested from the femurs and tibias of mice. Murine bone marrow cells were cultured for 18 h in tissue culture dishes in complete MEM-α media (Life Technologies, Grand Island, NY, USA) containing 10 % fetal bovine serum (FBS), 100 U/mL penicillin, and 100 μg/mL streptomycin to remove adherent cells. To allow bone marrow progenitor cells to differentiate into BMMs, non-adherent cells were transferred to new tissue culture dishes and cultured for 7 days in complete MEM-α media supplemented with 50 ng/mL murine recombinant M-CSF (R&D systems, Minneapolis, MN, USA). After seven days, the suspended cells were discarded and the attached BMMs were plated in tissue culture dishes. FTY720 was obtained from Santa Cruz biotechnology (Dallas, TX, USA), diluted in ethanol (10 mM), and stored at - 20 °C.

### Culture of *A. actinomycetemcomitans*

*A. actinomycetemcomitans* (ATCC 43718, serotype b, strain Y4) was purchased from American Type Culture Collection (Manassas, VA, USA). Bacterial colonies were grown on Difco™ brain heart infusion agar plates (BD Biosciences, Sparks, MD, USA) and cultured in Bacto™ brain heart infusion broth (BD Biosciences) at 37 °C with 10 % CO_2_ for 24 h. Bacteria were centrifuged, washed with PBS with 5 % glycerol, and resuspended in PBS with 5 % glycerol. Bacterial concentration was determined by measuring bacterial optical density and followed by plating on brain heart infusion agar plates (OD_600_ = 1, about 3 × 10^7^ colony forming unit, CFU/mL).

### Enzyme-linked immunosorbent assay (ELISA)

IL-1β, IL-6, and TNF-α cytokine levels in the cell culture media of BMMs were quantified by ELISA kits (R& D Systems). The protein concentrations from cell lysates were determined by a DC protein Assay Kit (Bio-Rad Laboratories, Hercules, CA, USA). The concentration of cytokines was normalized by protein concentration in cell lysates.

### Cell viability assay

Bone marrow cells (1 × 10^5^/well) in a 96-well plate were incubated with either vehicle (ethanol) or FTY720 (2 to 8 μM) for 8 to 24 h. The cell viability was analyzed by CellTiter 96 Aqueous One Solution Cell Proliferation Assay (Promega, Madison, WI, USA).

### Western blot assay

BMMs were lysed in RIPA protein lysis buffer (Cell Signaling Technology, Danvers, MA, USA). Western blot was performed as previously described [37]. The antibodies to p-PI3K, PI3K, p-Akt, Akt, p-ERK, ERK, p-JNK, p-p38, p-NF-κBp65, and GAPDH were purchased from Cell Signaling Technology (Danvers, MA, USA).

### Osteoclastogenesis assay and tartrate-resistant acid phosphatase (TRAP) staining

Murine bone marrow cells were cultured for 18 h in tissue culture dishes in complete MEM-α media to remove adherent cells. Non-adherent cells were transferred to new tissue culture dishes and cultured for 2 days in complete MEM-α media, supplemented with 50 ng/mL murine recombinant M-CSF to allow cells to differentiate into pre-osteoclasts. At day 3, cells were plated at a density of 1×10^6^ cells/well in 12-well plates or 1.5 × 10^5^ cells/well in 96-well plate, and cultured in complete MEM-α media, supplemented with either 50 ng/mL murine recombinant M-CSF alone (control) or with both 50 ng/mL M-CSF and 100 ng/mL RANKL (R&D Systems). At day 6, the media were changed and the cells were cultured in fresh MEM-α media containing 50 ng/mL M-CSF with or without 100 ng/mL RANKL. The cells were treated with either FTY720 (2 μM) or vehicle (ethanol) for 30 min. Then the cells were either unstimulated or stimulated with *A. actinomycetemcomitans* (0.5 CFU/cell) in the presence of FTY720 or vehicle for 4 h or for 24 h. Control cells were treated with M-CSF alone with or without bacterial stimulation. TRAP staining was performed in the cells using a leukocyte acid phosphatase kit (Sigma Aldrich, St. Louis, MO, USA) 24 h after FTY720 treatment. Pictures were taken by a Nikon Eclipse TS-100 inverted microscope. Image analysis was performed using Visiopharm 5.0 software (Visiopharm, Hoersholm, Denmark).

### RNA extraction and real time polymerase chain reaction (PCR)

The RNA extraction, reverse transcription, and real time PCR were performed as previously described [2]. The following amplicon primers were obtained from Life Technologies: Nfatc1 (Mm00479445_m1), Ctsk (Mm00484039_m1), Acp5 (Mm00475698_m1), Oscar (Mm00558665_m1), RANKL (Mm00441906_m1), and GAPDH (Mm99999915_g1). Amplicon concentration was determined using threshold cycle values compared with standard curves for each primer. Sample mRNA levels were normalized to an endogenous control GAPDH expression and expressed as fold changes compared with control groups.

### Statistical analyses

All experiments were performed in triplicate with bone marrow cells from mice. Data were analyzed by one-way ANOVA with Holm-Sidak’s multiple comparisons test. All statistical tests were performed using GraphPad Prism software (GraphPad Software Inc., La Jolla CA, USA). Values are expressed as mean ± standard error of the mean (SEM). A *P* value of 0.05 or less was considered significant.

## Abbreviations

*A. actinomycetemcomitans*: *Aggregatibacter actinomycetemcomitans*
Acp5: Acid phosphatase 5
BMM: Bone marrow-derived monocytes and macrophages
CFU: Colony forming unit
Ctsk: Cathepsin K
ELISA: Enzyme-linked immunosorbent assay
ERK: Extracellular signal-regulated kinase
FBS: Fetal bovine serum
GAPDH: Glyceraldehyde 3-phosphate dehydrogenase
IL: Interleukin
JNK: c-Jun N-terminal kinase
MAPK: Mitogen-activated protein kinase
Nfatc1: Nuclear factor of activated T-cells cytoplasmic calcineurin-dependent 1
M-CSF: Macrophage colony-stimulating factor, NF-κB, nuclear factor-kappaB
Oscar: Osteoclast-associated receptor
PCR: Polymerase chain reaction
PI3K: Phosphoinositide 3-kinase
RANKL: Receptor activator of nuclear factor kappa-B ligand
SEM: Standard error of the mean
S1P: Sphingosine-1-phosphate
S1PR: Sphingosine-1-phosphate receptor
TRAP: Tartrate-resistant acid phosphatase
